# Undiagnosed Hemoglobinopathies: A potential threat to the premarital screening program

**DOI:** 10.12669/pjms.35.6.976

**Published:** 2019

**Authors:** Hassan A. Hamali, Muhammad Saboor

**Affiliations:** 1 Hassan A. Hamali, Ph.D, Medical Laboratory Technology Department, Faculty of Applied Medical Sciences, Jazan University, Jazan, Saudi Arabia; 2 Muhammad Saboor, Ph.D, Medical Laboratory Technology Department, Faculty of Applied Medical Sciences, Jazan University, Jazan, Saudi Arabia

**Keywords:** Premarital screening, thalassemia, sickle cell anemia, hemoglobinopathy

## Abstract

**Objectives:**

To evaluate the prevalence of undiagnosed hemoglobinopathies among individuals visiting the premarital screening Centre.

**Methods:**

This study was conducted at Premarital Screening Centre, King Fahad Central Hospital and Research Centre, Jazan, between January 2018 and October 2018. A total of 3,970 (male n =1,859 and female n = 2,111) individuals were included in the study. Data of complete blood count, hemoglobin electrophoresis and sickling tests of all individuals recruited in the study were obtained and statistically analyzed.

**Results:**

One thousand three hundred and twelve individuals had abnormal complete blood counts or hemoglobin electrophoresis results, that include sickle cell trait (13.5%), sickle cell disease (0.7%), β thalassemia with sickle cell trait (2.46%), β thalassemia trait (1.51%), β thalassemia major (0.075%), suspected α thalassemia or other hemoglobinopathies (4.43%), hemoglobin H (0.3%), hemoglobin E (0.075%), undiagnosed cases (0.91%) and iron deficient (7.23%).

**Conclusion:**

A high percentage of individuals are suspected for α thalassemia or other hemoglobinopathies that needs to be diagnosed. Further investigations shall be included in the premarital screening program to diagnose these inconclusive cases. Coexistence iron deficiency with thalassemia shall also be ruled out during premarital screening program.

## INTRODUCTION

Before marriage, couples are tested for various hereditary, infectious or blood borne diseases that may be transmitted from them to their offspring, through a process known as premarital screening. Incidence of genetic disorders in the Middle East is most common including thalassemia and sickle cell anemia.^1^,^2^ In 2004, third Royal Decree mandated the premarital screening of thalassemia and sickle cell anemia. According to this decree, couple getting married, has to obtain a certificate from the authorized health centers, after being tested, stating their thalassemia and sickle cell anemia status. Counseling of the couples at risk is another important task of this program.

Some authors found the increased rate of marriage cancellation due to better understanding of the disease and counseling by the couples at risk.^3^,^4^ However, the actual targets have not been achieved yet due a number of reasons including consanguineous marriages, late stage screening (just before the marriage), personal or familial commitment, non-availability of alternative suitable partner anddeclaration of carrier status of the female.^5^,^6^ Other contributory factors may include β thalassemia with concomitant iron deficiency anemia, lack of molecular testing, α thalassemia and other variants of β thalassemia.

The aim of this study was to audit the individuals undergoing premarital screening for hemoglobinopathies and undiagnosed cases with suggestive results of thalassemia or its variants. The study hypothesized that undiagnosed cases of hemoglobinopathies play a role in the propagation of these disorders.

## METHODS

This study was conducted at Premarital Screening Centre, King Fahad Central Hospital and Research Centre, Jazan, between January 2018 and October 2018. A total of 3,970 (male n =1,859 and female n = 2,111) individuals were recruited in the study. Ethylene diamine tetra acetic acid anticoagulated tubes containing blood samples were run on Sysmex XN–1000 (Japan) for complete blood count. Hemoglobin electrophoresis was performed using Variant II Hemoglobin Testing System. Hemoglobin electrophoresis results that were ambiguous, inconclusive or needed separation of hemoglobin S and hemoglobin D^Punjab^ (S/D) and hemoglobin A_2_, hemoglobin C, and hemoglobin O^Arab^ (A_2_/C/E/O) A_2_/C/E/O were tested using Sebia capillary electrophoresis system. Data of complete blood count and hemoglobin electrophoresis of all employed individuals were obtained. To maintain confidentially of the volunteers as per agreement, no demographic data were taken out from the premarital center.

### Ethical Approval

The study was approved (Ref.No. REC39/4-348 dated on December 11, 2017) by Scientific Research Ethics Committee–Jazan University.

### Inclusion Criteria

All adult individuals undergoing premarital screening at the screening center who gave informed consent were recruited in the study.

### Exclusion Criteria

Individuals having a recent history of systemic disorders and other hematological disorders were excluded from the study.

### Statistical Analysis

Data were analyzed using Statistical Package for the Social Sciences (SPSS) software, version 17.0 for Windows (SPSS for Windows, Chicago, IL, USA). An independent samples t-test and one way ANOVA were used to compare variables where appropriate. A p value < 0.05 was accepted as statistically significant.

## RESULTS

A total of 3,970 (male n =1,859 and female n = 2,111) individuals were included in the study fulfilling the inclusion criteria. Based upon the analytical laboratory data, subjects were divided into twelve groups as shown in Table-I. Individuals with abnormal complete blood counts or hemoglobin electrophoresis were 1,312 (33.04%). Sickle cell trait was the most common hemoglobinopathy among the studied population (Table-I). Individuals suspected for α alpha thalassemia or other variants of thalassemia were found to be 176 (4.43%) which is also highly prevalent. A small number of individuals (n=36, 0.90%) showed statistically high hemoglobin F levels (p<0.0001) as compared to control groups. These individuals were labeled as normal although having high hemoglobin F levels. Referring to Table-I, 287 (7.22%) female subjects were found iron deficient. Red cell parameters and hemoglobin electrophoresis results of male and female groups are given in Table-II.

**Table I:** Prevalence of hemoglobinopathies and other conditions in the studied population.

Groups	Number of subjects	Percentage
Normal	2,658	66.95
Sickle cell trait	536	13.50
Sickle cell disease	28	0.70
β thalassemia with sickle cell	98	2.46
β thalassemia trait	60	1.51
β thalassemia major	03	0.075
α thalassemia (suspected) or variants of thalassemia	176	4.43
α thalassemia (suspected) with sickle cell trait	73	1.83
Hemoglobin H disease	12	0.30
Hemoglobin E	03	0.075
Undiagnosed	36	0.90
Anemia	287	7.22

Total	3,970	100

**Table II:** Red cell parameters and hemoglobin electrophoresis of male and female groups.

Rubrics	Groups	RBC	Hb.	Hb. A	Hb. A_2_	Hb. F	Hb. S
Normal	M	5.33±0.38	14.81±1.71	97.04±0.19	2.91±0.23	0	0
	F	4.83±0.43	12.92±1.00	97.13±0.36	2.03±0.36	0	0
α thalassemia	M	6.35±0.48	14.19±1.09	97.7±0.50	2.25±0.44	0	0
	F	5.97±0.49	12.55±1.00	97.75±0.42	2.21±0.40	0	0
β thalassemia	M	6.41±0.40	13.15±0.99	93.93±0.93	5.68±0.63	0	0
	F	5.77±0.67	11.03±0.81	93.75±1.05	5.41±0.41	0	0
β thalassemia with sickle cell trait	M	5.48±1.49	12.21±2.78	67.14±6.62	4.74±0.80	4.74±0.80	27.78±7.57
	F	5.23±0.24	10.95±0.70	69.88±0.79	3.93±0.15	3.93±0.15	26.11±6.3
Sickle cell trait	M	5.59±1.17	14.44±2.07	60.26±3.29	3.01±0.06	3.01±0.06	36.63±3.21
	F	4.7±0.80	11.41±1.32	63.44±3.68	3.02±0.16	3.02±0.16	33.50±3.74
Sickle cell trait with α thalassemia or other variants	M	6.1±0.61	13.25±1.2	59.56±1.2	3.3±0.06	1.8±0.40	35±4.5
	F	5.5±0.50	10. ±0.90	61. ±0.85	3.1±0.67	2.4±0.79	34±3.9
Undiagnosed	M	4.77±0.85	12.5±1.80	95.11±0.83	2.83±0.21	2.05±0.88	0
	F	4.75±0.85	12.50±1.80	95.11±0.83	2.83±0.21	2.15±0.98	0
Iron deficient	M	0	0	0	0	0	0
	F	4.79±0.84	10.16±1.05	97.77±0.42	2.21±0.41	0	0

M= male, F= female, RBC= Red cell count (x10^12^/L), Hb = hemoglobin (g/dl).

## DISCUSSION

Premarital screening program for hereditary disorders and acquired viral diseases is of vital importance as it dictates the prevalence of these disorders and their possible transmission to the offspring. Consanguineous marriages are most common in the Kingdom of Saudi Arabia that plays instrumental role in the propagation of hereditary disorders. Premarital screening program helps convince the future couples with trait status for hemoglobinopathies not to marry due to the disastrous outcome in the form of children with diseased status i.e. sickle cell disease and thalassemia major.

The most common abnormal finding observed in the studied population was sickle cell trait (13.52%). A large scale study conducted across the kingdom based upon various premarital centers has shown the highest prevalence of sickle cell anemia in the Eastern and Jazan regions.^7^ Another study conducted by Memish et al also reported that the prevalence of sickle cell trait was highest in the Eastern region and Jazan region (13.41% and 12.66% respectively).^8^

It may be also noted that 0.71% patients with sickle cell disease also visited the screening center to seek the premarital screening certificate. The reported overall prevalence of sickle cell disease was 0.38% in a previous study.^7^ A study conducted by Al- Saeed et al^9^ whom aim was to detect hemoglobinopathies in adult patients, reported the prevalence of sickle cell trait to be 8.8%.^9^ These results showed much higher prevalence than reported in any other study, however, the reason for this high incidence may be the targeted anemic patients. A study, conducted in Jeddah (Western region), has shown that almost 11.2% of the general population in this area is trait for abnormal genes; sickle cell trait being the highest i.e. 5.4%.^10^ Data reported from Al-Baha region has shown the prevalence of sickle cell trait to be 3.7% and 0.99% for sickle cell disease.^11^ Prevalence of sickle cell trait is Al-Baha region is much lower as compared to Jazan. Analysis of all the above mentioned studies reveals that sickle cell trait is the most prevalent hemoglobinopathy in Jazan as compared to other regions of the Kingdom.

Individuals with β thalassemia trait were found to be 1.51% in this study. These findings are similar to other studies conducted in the kingdom. The overall prevalence of β thalassemia in the kingdom was found to be 1.29%^7^, 1.80%^8^ and 1.3%^11^ in different studies and different geographical distribution within the Kingdom. These studies suggest almost similar distribution of β thalassemia in all parts of the kingdom except the Eastern region (5.9%).^8^ Studies, conducted in Jeddah (West region) and Al-Hassa (East region), have shown that 4.69% and 3.4% individuals had β thalassemia trait which is very high as compared to the findings of the current study.^10^,^12^

β thalassemia with sickle cell trait was also found in 2.47% individuals in the studied population. This finding is contrary to results of other studies where the reported prevalence of β thalassemia with sickle cell trait to be 3.3% and 3.4%.^9^,^12^

It may be noted that a high percentage (4.43%) of the studied population was labeled as “suspected for α thalassemia”. No definite diagnosis was made for these subjects. Suspicion for α thalassemia was made on the basis of subnormal hemoglobin levels, significantly high red cell count, low mean cell hemoglobin and almost normal mean cell hemoglobin concentration. From laboratory point of view, the technical reason of non-diagnosis of these cases is the inability of hemoglobin electrophoresis to detect α thalassemia. Secondly, some β thalassemia variants also show normal hemoglobin electrophoresis results with subnormal or borderline hemoglobin, high red cell count, slightly lower mean cell hemoglobin and almost normal red cell distribution width. Care should be taken while labeling them as α thalassemia. Earlier studies conducted in the Kingdom have shown high prevalence of α thalassemia in Eastern region as compared to any other region of Saudi Arabia.^13^ However, it may be noted that data regarding α thalassemia is very limited. For successful premarital screening program that have to minimize the transfer of hereditary disorders, the evaluation of α thalassemia may be made mandatory. Sharma et al reported that out of 54 microcytic anemia subjects, 19 (35.2%) had α thalassemia mutations showing high prevalence of α thalassemia in microcytic anemias.^14^ Another study has shown that 50% of non-anemic subjects with microcytosis had α thalassemia.^15^ The findings of this study also showed that 0.30% of the tested individuals had Hemoglobin H disease; a subtype of α thalassemia. This finding indicates the presence of α thalassemia gene in this area. A study by Quadri and Islam has shown that Hemoglobin H disease is the frequently encountered disease in the Dammam region.^13^ It is highly recommended that molecular analysis for α thalassemia must be employed to exclude α thalassemia. It was also noted that 1.83% individuals with sickle cell trait were also positive for α thalassemia or other variants of thalassemia. These cases also need attention.

A small proportion (0.075%) of the studied population was found to have hemoglobin E. The prevalence of hemoglobin E reported in Jeddah was 0.85% that is much higher than the current study.^10^ Data about hemoglobin E is also scanty.

One of the most significant finding of this study was individuals with high hemoglobin F as compared to normal control groups as shown in Table-II. This group was labeled as undiagnosed due to normal complete blood counts despite high hemoglobin F. Studies have shown that hemoglobin F may be slightly increased in various hemoglobinopathies including δβ thalassemia, rare cases of β thalassemia trait with thalassemia including coexisting β and δ thalassemia and coexisting β and α thalassemia, α thalassemia trait and α or δ globin chain variant.^16^ A large scale study based on genetic analysis is highly recommended to rule out the presence of these hemoglobinopathies.

Significant numbers of females were found to have iron deficiency (7.23%). Prevalence of iron deficiency in females is most common hematological disorder across the globe. It may be noted that we did not find any case of iron deficiency anemia concomitant with β thalassemia trait. It may be due to the low prevalence of β thalassemia in the local population. Contradictory reports are available in literature showing variable findings of hemoglobin A_2_ in individuals with iron deficiency anemia concomitant with β thalassemia trait. A study, conducted in India, reported that iron deficiency concomitant with β thalassemia trait was very unlikely to significantly affect the levels of hemoglobin A_2_ in severe iron deficient females with concomitant β thalassemia trait.^17^ A study by Usman et al has shown that individuals with β thalassemia trait coexistent with iron deficiency anemia had normal hemoglobin A_2_ levels that were confirmed by molecular analysis.^18^ Iron supplementation for 20 weeks normalized their iron level and significant changes were also observed in these individuals. Another study conducted by Verma et al also reported the same results with significant increase in the level of serum iron, ferritin, and hemoglobin A_2_ levels in iron deficient subjects with coexistent β thalassemia trait after supplementation.^19^ As individuals with β thalassemia trait have increased levels of hemoglobin A_2_ while iron deficiency reduces it, it has been suggested that care shall be taken while dealing with such cases. From the above results and discussion it is concluded that screening for α thalassemia using molecular techniques shall be incorporated in the premarital screening program. Silent mutations of thalassemia indicative of thalassemia based upon complete blood count with normal hemoglobin electrophoresis shall be ruled out by molecular studies. Coexistence iron deficiency with thalassemia shall also be ruled out during premarital screening program.

### Authors’ Contribution:

**HAH:** Designed the project and reviewed the manuscript.

**MS:** Collected the data and wrote the manuscript.
